# *Rhus coriaria* suppresses angiogenesis, metastasis and tumor growth of breast cancer through inhibition of STAT3, NFκB and nitric oxide pathways

**DOI:** 10.1038/srep21144

**Published:** 2016-02-18

**Authors:** Hussain El Hasasna, Alaaeldin Saleh, Halima Al Samri, Khawlah Athamneh, Samir Attoub, Kholoud Arafat, Nehla Benhalilou, Sofyan Alyan, Jean Viallet, Yusra Al Dhaheri, Ali Eid, Rabah Iratni

**Affiliations:** 1 Department of Biology, College of Science, UAE University, United Arab Emirates University, Al-Ain, P.O. Box 15551, United Arab Emirates; 2 Department of Biological and Environmental Sciences, College of Arts and Sciences, Qatar University, P.O. Box: 2713, Doha, Qatar; 3 Department of Pharmacology & Therapeutics, College of Medicine & Health Sciences, United Arab Emirates University, Al-Ain, P.O. Box: 17666, United Arab Emirates; 4 INSERM U823, Université Joseph Fourier, BP170, 38042 Grenoble, France; 5 Department of Pharmacology and Toxicology, Faculty of Medicine, American University of Beirut, Beirut, PO Box 11-0236, Lebanon; 6 Department of Biological and Environmental Sciences, College of Arts and Sciences, Qatar University, PO Box: 2713, Doha, Qatar

## Abstract

Recently, we reported that *Rhus coriaria* exhibits anticancer activities by promoting cell cycle arrest and autophagic cell death of the metastatic triple negative MDA-MB-231 breast cancer cells. Here, we investigated the effect of *Rhus coriaria* on the migration, invasion, metastasis and tumor growth of TNBC cells. Our current study revealed that non-cytotoxic concentrations of *Rhus coriaria* significantly inhibited migration and invasion, blocked adhesion to fibronectin and downregulated MMP-9 and prostaglandin E_2_ (PgE2). Not only did *Rhus coriaria* decrease their adhesion to HUVECs and to lung microvascular endothelial (HMVEC-L) cells, but it also inhibited the transendothelial migration of MDA-MB-231 cells through TNF-α-activated HUVECs. Furthermore, we found that *Rhus coriaria* inhibited angiogenesis, reduced VEGF production in both MDA-MB-231 and HUVECs and downregulated the inflammatory cytokines TNF-α, IL-6 and IL-8. The underlying mechanism for *Rhus coriaria* effects appears to be through inhibiting NFκB, STAT3 and nitric oxide (NO) pathways. Most importantly, by using chick embryo tumor growth assay, we showed that *Rhus coriaria* suppressed tumor growth and metastasis *in vivo*. The results described in the present study identify *Rhus coriaria* as a promising chemopreventive and therapeutic candidate that modulate triple negative breast cancer growth and metastasis.

Breast cancer remains one of the most common cancers as well as one of the leading causes of worldwide cancer-related morbidity and mortality^1^. Conventional treatment for breast cancer includes radiation, surgery and chemotherapy. Despite the fact that these treatment modalities have improved over the last decade, prognosis of this cancer remains poor. Common chemotherapeutic agents target three receptors: estrogen receptor (ER), progesterone receptor (PR), and human epidermal growth factor receptor (HER2)^2^. Breast cancer cells that lack ER, PR and show low HER2 levels are often referred to as triple negative breast cancer (TNBC)^3^. In addition to lacking the three receptors, most TNBC cells evade apoptosis and avoid DNA repair mechanisms^4^. Patients diagnosed with TNBC tend to have increased recurrence and mortality rate within 5 years of cancer detection and are thus considered to have poorer prognosis^5^,^6^. Even with combination treatment regimens, survival rates of this highly metastatic type of cancer remain low. As such, novel compounds that could be more effective with potentially less side effects are critically needed.

Alternative approaches like herbal medicine have been sought and given more attention in recent years. Contextually, a large percent of women appear to be highly interested in using herbal remedies to prevent or treat breast cancer^7^. This increased interest is likely due to the fact that herbs and plants contain a bountiful presence of bioactive compounds such as: flavonoids, alkaloids and steroids in herbs and plants^8^. Indeed, most of the currently employed chemotherapeutic agents have been generated from or inspired by natural products. For example, during the last three decades alone, only 20% of all new chemical entities introduced as drugs are classified as “synthetic”^9^. On the contrary, the majority of all cancer chemotherapeutic agents introduced since 1940 are either natural products, directly derived therefrom, or mimic one of these products^9^.

Among the different herbs included in the pharmacopeias of many countries especially in the Levant, the Rhus genus is known for its medicinal values^10^,^11^. This genus comprises more than 250 different species of flowering plants in the family Anacardiaceae^10^. Of particular interest is the species known as *Rhus coriaria* L., which is commonly known as sumac. Indeed, of all the Rhus species, *Rhus coriaria* is the most widely consumed species in the Mediterranean region^12^. Interestingly, the use of *Rhus coriaria* fruits in different countries on the rise^13^.

*Rhus coriaria* grows as a small tree with a height range of 1–4 meters in the wild^14^. The dried fruit of this plant is the most commonly consumed part and is typically used as a condiment, owing to its sour taste. *Rhus coriaria* has been shown to exhibit antimicrobial, anti-inflammatory, anti-fibrogenic, antifungal and anti-atherogenic properties^10^,^11^. Some of these capacities have been attributed to *Rhus coriaria*’s phenolic acids, flavonoids and tannins^11^,^15^,^16^,^17^. We have recently shown that ethanolic extract of *Rhus coriaria* induces cell cycle arrest along with concomitant autophagic cell death of TNBCs^18^. However, whether it can modulate the metastatic phenotype of these cells remained largely obscure. Here, we sought to determine the effect of *Rhus coriaria* on the malignant behavior of MDA-MB-231 cells and determine the underlying mechanisms. Our results indicate that *Rhus coriaria* extract (RCE) abolishes the migration, invasion of TNBCs, suppresses angiogenesis and reduces tumor growth *in vivo* via inhibition of STAT3, NFκB and nitric oxide (NO) pathways.

## Materials and Methods

### Cell culture and reagents

Human breast cancer cells MDA-MB-231(cat# 300275) were obtained from Cell line service (CLS)-GmbH. MDA-MB-231-GFP was previously described^19^. MDA-MB-231and MDA-MB-231-GFP were maintained in DMEM (Hyclone, Cramlington, UK). All media were complemented with 10% fetal bovine serum (FBS) (Hyclone, Cramlington, UK), 100 U/ml penicillin/streptomycin (Hyclone, Cramlington, UK). Human Umbilical Vein Endothelial Cells (HUVECs) (Cat # C-003-5C) were obtained from (Life Technologies, Invitrogen). HUVECs were maintained in MEM 199 supplemented with 20% FBS, penicillin/streptomycin, 2 mM L-glutamine, 5 U/ml heparin and 50 μg/ml endothelial cell growth supplements (BD Biosciences, Bedfrord, MA, USA). Human Lung Microvascular Endothelial Cells (HMVEC-L) (cat # CC-2527) were obtained from Lonza (Lonza, Walkersville, USA). HMVEC-L cells were maintained in EGM™-2-MV BulletKit (Lonza, Walkersville, USA). Antibodies to NF-κB, phospho-p65, TNF-α and iNOS were obtained from Abcam. Antibodies to STAT3, pSTAT3, β-actin, goat anti-mouse IgG-HRP (sc-2005) and goat anti-rabbit IgG-HRP (sc-2004) were obtained from Santa Cruz Biotechnology, Inc. LY294002 was purchased from Sigma-Aldrich.

### Preparation of the *Rhus coriaria* Ethanolic Extract (RCE)

Fruits of *Rhus coriaria L.* were collected from a private farm located at 33° 16′ 35.59″ N and 35° 19′ 02.89″ E. The farm is located in Ma’rakeh, Tyre, Lebanon and the approval of the owner was obtained before collecting the fruit or commencing any experiments. This plant is neither endangered nor protected by any laws and it is readily and commercially available in the market. RCE was prepared as previously described^18^. Briefly 10.0 g of the dried fruit were ground to a fine powder using a porcelain mortar and pestle. The powder was then suspended in 100 mL of 70% absolute ethanol and the mixture was kept in the dark for 72 hours at 4 °C in a refrigerator without stirring. After that, the mixture was then filtered through a glass sintered funnel and the filtrate was evaporated to dryness using a rota-vapor at room temperature. The red residue was kept under vacuum for 2–3 hours and its mass was recorded. The residue was stored at −20 °C until further use.

### Matrigel invasion assays

Invasiveness of the MDA-MB-231 cells treated with the RCE was tested using BD Matrigel Invasion Chamber (8-μm pore size; BD Biosciences, Bedfrord, MA, USA) according to manufacturer’s instructions. Briefly, MDA-MB-231cells (1 × 10^5^ /well) were placed in 0.5 mL of media containing vehicle (0.2% ethanol) or the indicated concentrations of RCE were seeded into the upper chambers of the system; the bottom wells in the system were filled with DMEM complemented with 10% fetal bovine serum as a chemo-attractant and then incubated at 37 °C for 24 h. Non-penetrating cells were removed from the upper surface of the filter with a cotton swab. Cells that have migrated though the matrigel were fixed with 4% formaldehyde, stained with DAPI. Fluorescence from DAPI was detected using a filter with excitation wavelength of 330–380 nm and barrier filter of 400 nm. DAPI-stained nuclei were counted in 10 random fields per well using inverted fluorescence microscope at X200 magnification (Nikon Ti-U, Nikon). For quantification, the assay was done in duplicates and repeated three times.

### Wound healing migration assay

Briefly, MDA-MB-231 cells were grown in six-well tissue culture dishes until confluency. A scrape was made through the confluent monolayer with a sterile plastic pipette tip. Three wounds were made for each sample. Afterwards, the plates were washed twice with PBS and incubated at 37 °C in fresh DMEM supplemented with 10% fetal bovine serum in the presence of vehicle (ethanol) or indicated concentrations of RCE. At the bottom side of each dish, three arbitrary places were marked where the width of the wound was photographed with an inverted microscope at X40 magnification (Nikon Ti-U, Nikon). Wound closure was determined by measuring the distance (μm) between the edges of the wound at time 0, 6 and 10 h, using the NIS-Elements BR 3.0 software (Nikon). Quantification of the distance migrated by the cells was done as follow: D = (Size of the wound at t = 0 h –size of the wound at t = 6 or 10 h).

### Migration Chamber Assay

MDA-MB-231 cells (5 × 10^4^/well) were seeded in the upper chamber of an insert system (BD Biosciences, USA) in serum-free medium, with or without RCE and migration assay was carried out according to the manufacturer’s instructions. Serum-containing medium was added to the lower chamber to act as a chemotactic attractant. After 6 hours of incubation, cells were washed with PBS, fixed with formaldehyde, and fixed with methanol. Cells were then stained with 1% crystal violet for 10 minutes. After washing with PBS, cells from at least five different random fields were counted under an inverted microscope.

### Adhesion Assay

Adhesion of MDA-MB-231 to HUVECs or HMVEC-L was performed as previously described^20^,^21^. Briefly, MDA-MB-231 cells, transfected with *Renilla* luciferase, were loaded onto confluent monolayers of endothelial cells in the presence of vehicle (0.2% ethanol) or RCE. Where indicated, TNF-α (25 ng/ml) was added for 6 hours to stimulate HUVECs prior to the addition of MDA-MB-231 cells. After 1 hour, unattached cells were removed by gently washing the plates three times with PBS. Adherent cells were lysed using a luciferase lysis buffer (Promega, Madison, WI, USA) and, luminescence was measured using Glomax 20/20 luminometer (Promega). The relative adhesion of MDA-MB-231 to endothelial cells (HUVECs or HMVEC-L) was expressed as the percentage of luminescence compared to the controls.

### Adhesion to Fibronectin Assay

96-well plates were coated with fibronectin (dissolved in PBS) and incubated at 37 °C overnight. After that, plates were blocked with 3% Bovine Serum Albumin (BSA) for 3 hours at room temperature. MDA-MB-231 cells (5 × 10^3^/well) were then seeded in growth medium and incubated for 60 minutes in the humidified incubator (37 °C with 5% CO_2_). Cells were then washed 3 times with PBS to remove non-adherent cells. Attached cells were stained with crystal violet and observed under the microscope. At least five random fields were counted and the experiment repeated three times.

### Transendothelial Migration Assay

Transendothelial migration of MDA-MB-231 through HUVECs was performed as previously described^20^,^21^. Briefly, transwell filters were coated with collagen and allowed to dry overnight. HUVECs (2 × 10^5^/well) were then seeded onto the rehydrated membrane and allowed to grow until a confluent monolayer is formed. Where mentioned, TNF-α (25 ng/ml) was added for 6 hours to stimulate HUVECs. MDA-MB-231 cells (1 × 10^6^/well) were then loaded on top and incubated overnight in the absence or presence of RCE. Cells on the upper chamber were removed with a cotton swab, whereas MDA-MB-231 on the bottom were stained and quantified as previously reported^20^.

### Measurement of matrix metalloproteinases by ELISA

Cells were seeded in 6-well plates in the presence of vehicle (0.2% ethanol) or indicated concentrations of RCE for 24 hours. The conditioned medium was collected and the levels of secreted MMP-9 were determined using immunoassay kits (Abcam, Cambridge, UK) according to the manufacturer’s protocol. The optical density at 450 nm of each sample was measured using an AMP Platos R 496 microplate reader (AMP Diagnostics, Poland). The proteins present in the conditioned media were concentrated using the Amicon Ultra-15 protein purification and concentration column (Millipore) and protein concentration was assayed using the BCA protein assay kit (Thermo Scientific). Levels of MMP-9 were normalized to the total protein level in each sample. The assays were performed in triplicates and three independent experiments were performed. Data are presented as mean values ± SEM.

### Vascular tube formation assay

Assessment of *in vitro* capillary formation used Matrigel (Becton Dickinson, Le Pont de Claix, France). Matrigel is a squamous cell carcinoma basement membrane matrix composed primarily of collagen IV, laminin, entactin, and heparan sulfate proteoglycans. The Matrigel matrix was thawed, gently mixed to homogeneity using cooled pipettes, and diluted v/v with the EndoGRO™-MV-VEGF Complete Media Kit medium (Millipore, Temecula, CA, USA). Matrigel, 50 μl/well, supplemented with angiogenic peptides and other effectors was used to coat the wells of 96-well plates. The plate was then incubated for one hour at 37 °C to allow the matrix solution to solidify prior to treatment. HUVECs (at a density of about 4 × 10^4^ cells/well) and the indicated concentration of RCE were plated to each well and incubated for 8 h at 37 °C in 0.1 mL of EndoGRO™-MV-VEGF Complete Media Kit medium (Millipore, Temecula, CA, USA). Then, cells were photographed using an inverted phase contrast photomicroscope at X (40 × 1.6) magnification (Olympus 1 × 71, Olympus). The tubular network growth area was compared in control and inhibitor-treated Matrigel matrix. Tube formation was quantified by counting the length of tube-like structures formed in each well. The effect of RCE on viability of the HUVECs was determined using a CellTiter-Glo Luminescent Cell Viability assay (Promega Corporation, Madison, USA), as previously described^20^. LY294002 (LY), an inhibitor of PI3K/AKT pathway and inhibitor of vascular tube formation, was used as positive controls for angiogenesis inhibition in this assay.

### Transient transfection assay

MDA-MB-231 cells (1.5 × 10^5^/well) were seeded in 12-well plates the day before transfection. Cells were then transfected with the pGL4.32[luc2P/NFκB-RE/Hygro] expression plasmid (Promega, Madison, WI, USA) using Fugene HD (Promega, Madison, WI, USA) according to the manufacturer’s instructions. Briefly, 18 h post-transfection, cells were incubated for another 24 h in fresh complete media containing increasing concentrations of RCE. Luciferase activity was measured using Dual Luciferase Reporter Assay System (Promega, Madison, WI, USA). *Renilla* luciferase reporter was used as an internal control, to which firefly luciferase values were normalized. Experiments were repeated three times and the average of three means is represented ± SEM.

### Quantification of Nitrate/Nitrite production

The amount of Nitrate/Nitrite production was determined with a colorimetric ELISA kit (Cayman Chemical, Ann Arbor, Michigan, USA), which is based on the Griess reaction, according to the manufacturer’s instructions. The value of nitrate/nitrite presented is the total value measured in the presence of cells minus the value determined from the media alone in the absence of any growing cells. Assays were performed in triplicates and three independent experiments were performed. Data are presented as mean values ± SEM.

### Quantitative Immunoassay for Human Vascular Endothelial Growth Factor (VEGF)

Cells (1.5 × 10^5^/well) were seeded in 24-well plates overnight in serum-containing culture media and then, the media was replaced by serum-free media. Cells were treated with vehicle (ethanol) or indicated concentrations of RCE and the conditioned media was collected at 24 h. The level of VEGF therein was measured using a VEGF enzyme-linked immunosorbent assay kit (R&D Systems, Minneapolis, MN, USA) according to the manufacturer’s protocol. The optical density at 570 nm of each sample was measured using an AMP Platos R 496 microplate reader (AMP Diagnostics, Poland). The proteins present in the conditioned media were concentrated using the Amicon Ultra-0.5 protein purification and concentration column (Millipore) and protein concentration was assayed using the BCA protein assay kit (Thermo Scientific). Levels of VEGF were normalized to the total protein level in each sample. The assays were performed in triplicates and three independent experiments were performed. Data are presented as mean values ± SEM.

### ELISA quantification of cytokines

Cells (2 × 10^5^/well) were cultured in a 6-well plate overnight and then serum-starved for 24 h in the presence of vehicle (ethanol) or indicated concentrations of RCE. Levels of IL-6, −8, and −11 in the collected media were measured using ELISA quantification kit (R&D Systems, Minneapolis, MN, USA) according to the manufacturer’s instructions. For PgE2, culture supernatants were assayed using an ELISA kit from Cayman Chemical (MI, USA). The optical density of each sample was measured using an AMP Platos R 496 microplate reader (AMP Diagnostics, Poland). The proteins present in the conditioned media were concentrated using the Amicon Ultra-0.5 protein purification and concentration column (Millipore) and protein concentration was assayed using the BCA protein assay kit (Thermo Scientific). Levels of the cytokines/PgE2 were normalized to the total protein level in each sample. Assays were performed in triplicates and three independent experiments were performed. Data are presented as mean values ± SEM.

### Whole Cell extract and Western Blotting analysis

Cells (1.8 × 10^6^) were seeded in 100 mm culture dishes and cultured for 24 h before addition of RCE at the indicated concentrations. After incubation, cells were washed twice with ice-cold PBS, scraped, pelleted and lysed in RIPA buffer (Pierce) supplemented with protease inhibitor cocktail (Roche) and phosphatase inhibitor (Roche). After incubation for 30 min on ice, cell lysates were centrifuged at 14,000 rpm for 20 min at 4 °C. Protein concentration of lysates was determined by BCA protein assay kit (Thermo Scientific) and the lysates were adjusted with lysis buffer. Aliquots of 25 μg of total cell lysate were resolved onto 8–15% SDS-PAGE along with PageRuler Plus Prestained Protein Ladder (Thermo Scientific). Proteins were transferred to nitrocellulose membranes (Thermo Scientific) and blocked for 1 h at room temperature with 5% non-fat dry milk in TBST (TBS and 0.05% Tween 20). Incubation with specific primary antibodies was performed in blocking buffer overnight at 4 °C. Horseradish peroxidase-conjugated anti-IgG was used as secondary antibody. Immunoreactive bands were detected by ECL chemiluminescent substrate (Thermo Scientific). Where needed, membranes were stripped in Restore western blot stripping buffer (Thermo Scientific) according to the manufacturer’s instructions.

### Animal Studies

All animal experiments were conducted in accordance with the French guidelines on the use of animals in scientific investigations with the approval of the local Ethical committee, (Institut Albert Bonniot, Grenoble, France: licence protocol N° 381029 and B3851610001).

### Chick embryo tumor growth and metastasis assay

The chick embryo tumor growth assay (INOVOTION, Grenoble, France) was performed as previously described^20^,^22^. Briefly, Fertilized White Leghorn eggs, obtained from the Société Française de Production Agricole (SFPA, St. Brieuc, France), were incubated at 38 °C with 60% relative humidity for 10 days. According to the French legislation, no ethical approval is needed for scientific experimentations using oviparous embryos (decree n° 2013–118, February 1, 2013; art. R-214–88). At stage E10, the chorioallantoic membrane (CAM) was dropped by drilling a small hole though the eggshell into the air sac and a 1 cm^2^ window was cut in the eggshell above the CAM. Cultured MDA-MB-231-GFP were detached by trypsinization, washed with complete medium and suspended in serum free DMEM. A 50 μl inoculum of 1 × 10^6^ MDA-MB-231-GFP cells was added onto the CAM of each egg. Eggs were then randomized in 4 groups of 15 eggs (to get sufficient surviving embryos at the end of the experiments). One day later, tumors began to be detectable. They were then treated during 8 days, every two days (E11, E13, E15, E17), by dropping 100 μl of either RCE (50 or 150 μg/mL), colchicine (2 μM) or vehicle (0.02% ethanol in PBS onto the tumor. At E19 the upper portion of the CAM was removed, transferred in PBS and the tumors were then carefully cut away from normal CAM tissue and weighted. In parallel, a 1 cm^2^ portion of the lower CAM was collected to evaluate the number of nodules, containing GFP-expressing cells. The fluorescent nodule were visualized *in situ* using whole mounts of tissue fixed in 4% formaldehyde in PBS and flattened between a hollow glass slide and a thick coverslip. In order to number the nodule, a thorough and complete visual scan of the piece of the lower CAM was done using Leica Macrofluo fluorescent microscope (Leica Microsystems GmbH) equipped with GFP filter. Chick embryos were sacrificed by decapitation.

### Statistical analysis

All statistical analysis were performed using SPSS version 21 for PC (IBM Corp.). Data were reported as group mean ± SEM. The data were analyzed via one-way ANOVA. Significance for all statistical comparisons was set at p < 0.05.

## Results

### *Rhus coriaria* attenuates the migration ability of MDA-MB-231 breast cancer cells

**Figure 1:**
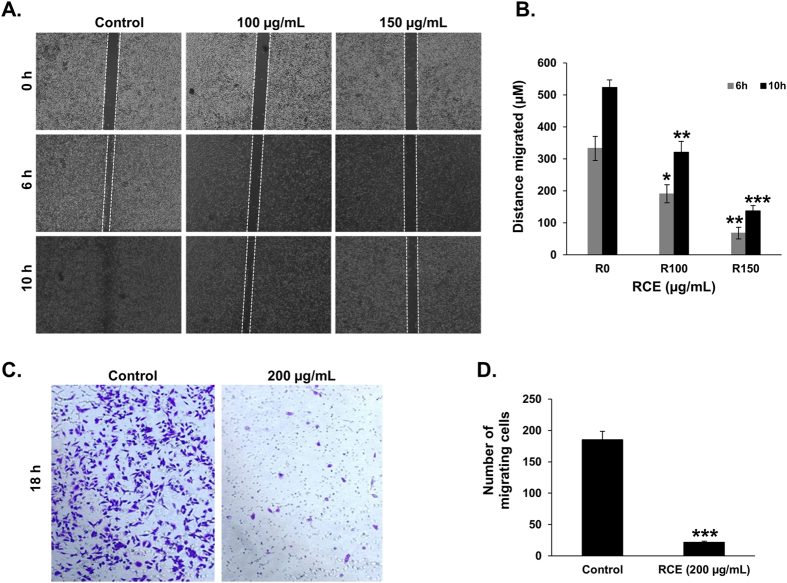
*Rhus coriaria* inhibits the migration of MDA-MB-231 human breast cancer cells. (**A**) Confluent culture of MDA-MB-231 cells were wounded by scratching with a pipette tip and the cells were incubated in DMEM supplemented with 10% fetal bovine serum without and with indicated concentrations of RCE. The wound was measured with an inverted microscope x 40 magnification and photographed. (**B**) Wound healing assay showing that RCE inhibited the migration of MDA-MB-231 breast cancer cells in concentration-dependent manner. Values represent the mean ± SEM (n = 3) distance (μm) that the cells have migrated in 6 and 10 h. Data are representative of three independent experiments. (**C**) Boyden chamber trans-well assay confirming that RCE inhibits the migration of MDA-MB-231 cells. Migrating MDA-MB-231 cells were stained with crystal violet and photographed with an inverted microscope at X100 magnification (Nikon Ti-U, Nikon). (**D**) Quantification of migrated MDA-MB-231 cells. The number of migrating MDA-MB-231 cells through Boyden chamber was quantified by counting at least 5 random fields at X200 magnification with an inverted microscope (Nikon Ti-U, Nikon). Data represent a mean ± SEM of cells counted per field and are representative of three independent experiments. Statistical analysis was performed using one-way ANOVA (*p < 0.05, **p < 0.005, ***p < *0.001*).

Because cell migration plays a crucial role in tumor metastasis, we first investigated the effect of RCE on the migration ability of MDA-MB-231 cells by using wound-healing migration assay. For this purpose, we performed the test with concentrations of RCE and periods of treatment that were non-cytotoxic to the MDA-MB-231 (Supplementary Figure 1). As shown in Fig. 1A and B, RCE treatment significantly inhibited cellular migration in a concentration-dependent manner. The ability of RCE to inhibit the migration of MDA-MB-231 cells was further confirmed using the Boyden chamber trans-well assay. As it is shown in Fig. 1C,D, the number of RCE (200 μg/mL)-treated cells that has migrated to the lower chamber was markedly reduced by approximately three fold when compared to vehicle (ethanol)-treated cells. Taken together, our data demonstrate that RCE inhibits the migration potential of MDA-MB-231 cells.

### *Rhus coriaria* inhibits the invasive potential of MDA-MB-231 cells, downregulates MMP-9 and prostaglandin E2, and reduces the adhesion of the breast cancer cells to fibronectin

**Figure 2:**
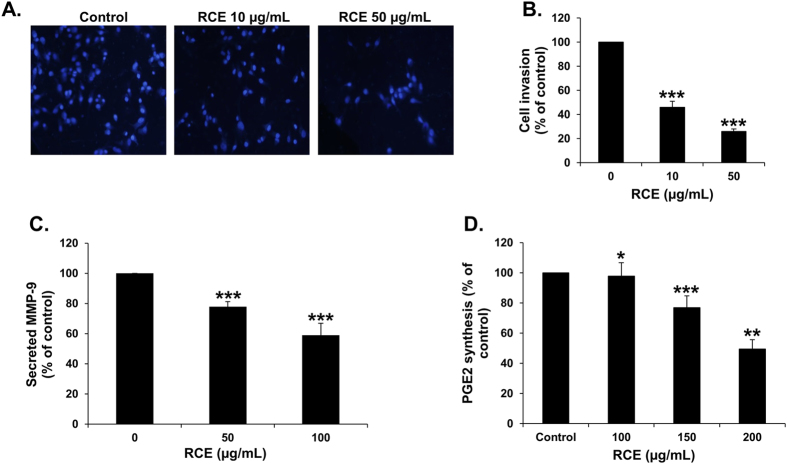
*Rhus coriaria* inhibits the invasion, downregulates MMP-9 and prostaglandin E_2_ (PgE2). (**A**) MDA-MB-231 cells were incubated for 24 h with or without RCE (10 and 50 μg/mL). Cells that invaded into the matrigel were scored as described in Materials and Methods. DAPI-stained nuclei of invading cells were photographed at X 100 magnification under an inverted microscope (Nikon Ti-U, Nikon). (**B**) Quantification of invaded MDA-MB-231 into the matrigel. Values represented in percent were calculated from three independent experiments and are represented as mean ± SEM. Statistical analysis was performed using one-way ANOVA (*p < 0.05, **p < 0.005, ***p < *0.001*). (**C**,**D**) Effects of RCE on the secretion of MMP-9 (**C**) and PgE2 (**D**) in RCE-treated MDA-MB-231 cells. The levels of secreted MMP-9 and PgE2 were determined using immunoassay kits as described in Materials and Methods. Experiments were repeated three times in triplicate and the average of three means is represented ± SEM. Statistical analysis was performed using one-way ANOVA (*p < 0.05, **p < 0.005, ***p < *0.001*).

Next, we examined the effect of RCE on the invasive potential of MDA-MB-231 cells in Matrigel-coated Boyden chamber in the presence of vehicle (ethanol) and RCE (10 and 50 μg/mL). The number of RCE-treated cells that has passed through the Matrigel coated membrane was markedly reduced in concentration-dependent manner by 60 and 80%, respectively (Fig. 2A,B), indicating that RCE efficiently inhibits the invasive capability of MDA-MB-231 cells.

Matrix metalloproteinase-9 (MMP-9), among other MMPs, is known to play an important role in breast cancer cell invasion and metastasis. To test whether *Rhus coriaria* inhibits breast cancer cell invasion by affecting the expression of MMP-9, we decided to examine the expression level of this protein in the conditioned medium using RCE-treated MDA-MB-231 cells. We found that secreted MMP-9 was significantly reduced in response to 50 and 100 μg/mL RCE treatment (Fig. 2C).

Prostaglandin E_2_ (PgE2) has been shown to be abundantly produced by various tumors, including breast cancer, and regulate cell growth, migration and invasion^23^,^24^. Here, we found that RCE could significantly reduce the level of PgE2 produced by the MDA-MB-231 breast cancer cells (Fig. 2D).

**Figure 3:**
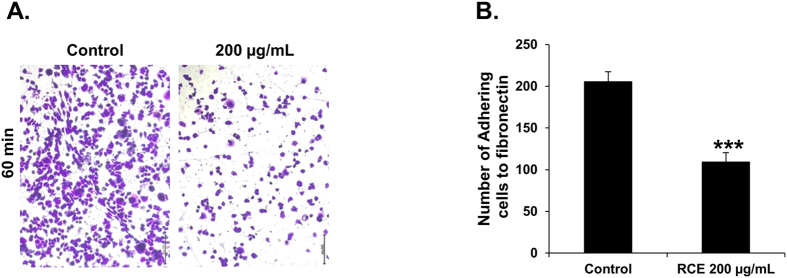
*Rhus coriaria* inhibits adhesion of MDA-MB-231 cells to fibronectin. (**A**) Effects of RCE on MDA-MB-231 cell adhesion to fibronectin-coated wells. MDA-MB-231 cells were seeded on the fibronectin-coated wells with or without RCE. After 60 min of incubation, non-adhered cells were washed. Attached cells were stained with crystal violet and photographed with an inverted microscope at X100 magnification (Nikon Ti-U, Nikon). (**B**) Quantification of attached MDA-MB-231 cells to fibronectin. The number of adherent MDA-MB-231 cells to fibronectin was determined by counting at least 5 random fields per well at X200 magnification with an inverted microscope (Nikon Ti-U, Nikon). Data represent a mean of cells counted per field and are representative of three independent experiments. Statistical analysis was performed using one-way ANOVA (***p < *0.001*).

Cell movement and adhesion of tumor cells to components of the extracellular matrix (ECM), such as fibronectin, and to basement membrane represent a crucial event in the process of cancer invasion and metastasis. Therefore, we also examined the effect of RCE on the ability of MDA-MB-231 to adhere to fibronectin. Interestingly, we found that RCE was able to inhibit MDA-MB-231 cells adhesion to fibronectin in a concentration-dependent manner (Fig. 3A,B). This effect was robust and fast as it appeared within the first 60 min of contact, at time at which no cell death occurred (data not shown).

Altogether, these findings suggest that downregulation of MMP-9 and PgE2, and impaired ability of cells to adhere to component of the extracellular matrix in response to RCE, account at least partly, in the inhibition of invasion of MDA-MB-231 cells.

### *Rhus coriaria* inhibits transendothelial migration of MDA-MB-231 through HUVECs and decreases their adhesion to HUVECs and to lung endothelial (HMVEC-L) cells

**Figure 4:**
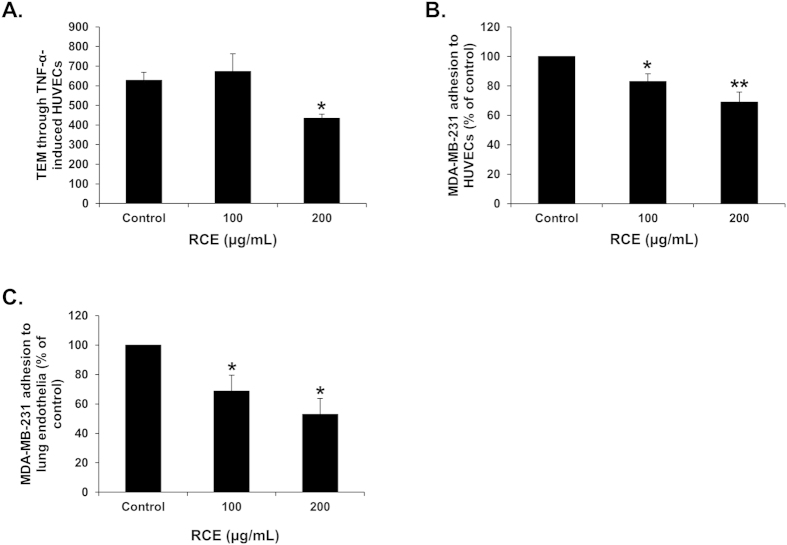
*Rhus coriaria* inhibits the migration of MDA-MB-231 cells across monolayer of TNF-α activated HUVECs and reduces their adhesion to HUVECs and lung endothelial cells (HMVEC-L). (**A**) Inhibition of transendothelial migration of MDA-MB-231 through TNF-α stimulated HUVECs. Confluent monolayers of HUVECs were pre-incubated with TNF-α (25 ng/mL) for 6 h, and transendothelial migration was evaluated after overnight incubation at 37 °C. Values represent means ± SEM, n = 3. Statistical analysis was performed using one-way ANOVA (*p < 0.05, **p < 0.005). (**B**,**C**) Inhibition of adhesion of MDA-MB-231 cells to HUVECs (**B**) and HMVEC-L (**C**). MDA-MB-231 cells were treated overnight with vehicle (0.2% ethanol) or indicated concentrations of RCE then seeded on top of the confluent endothelial cells and allowed to adhere for 60 min as described in Materials and Methods. Adhesion of MDA-MB-231 cell was represented as percentage of cells that have adhered to HUVECs (**B**) or HMVEC-L (**C**). Data represent means ± SEM of three independent experiments. Statistical analysis was performed using one-way ANOVA (*p < 0.05, **p < 0.005).

Migration of tumor cells through the vascular endothelium is a crucial event for metastasis, both during intravasation and extravasation. Here, we tested the ability of RCE-treated breast cancer cells to migrate through TNF-α stimulated HUVECs. As shown in Fig. 4A, transendothelial migration of RCE-treated MDA-MB-231 cells through the monolayer of HUVECs was significantly attenuated by *Rhus coriaria*.

Because the attachment of tumor cells to endothelial blood vessels is an early event required for transendothelial migration and hence for metastasis, we tested the effect of RCE on the ability of MDA-MB-231 cells to adhere to TNF-α stimulated HUVECs. As shown in Fig. 4B, RCE was able to inhibit, in a concentration-dependent manner, the adhesion of the cancer cells to HUVECs.

Lung is known to be a primary target for breast cancer metastasis. Therefore, we investigated the effect of RCE on the ability of MDA-MB-231 cells to attach to lung endothelial cells. Figure 4C shows that adhesion of MDA-MB-231 cells to pulmonary microvascular endothelial, HMVEC-L cells was also significantly inhibited by RCE, in a concentration-dependent manner (Fig. 4C).

### *Rhus coriaria* inhibits angiogenesis and suppresses VEGF production in HUVECs and MDA-MB-231 cells

**Figure 5:**
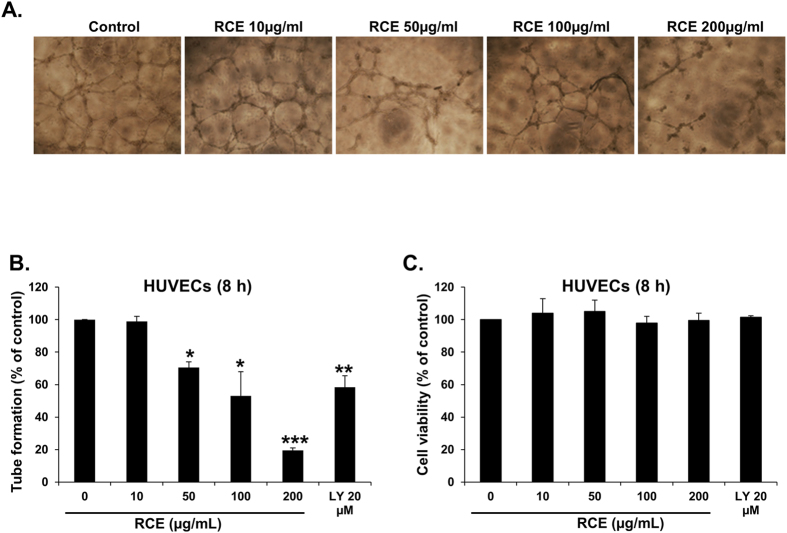
Effect of *Rhus coriaria* on the formation of capillary-like structures by HUVECs *in vitro.* (**A**) Patterns of angiogenesis induced by human umbilical vein endothelial cells (HUVECs) cultured on Matrigel matrix in 96-well plates with or without RCE. (**B**) Quantification of tubular morphogenesis induced in HUVECs cells cultured with or without RCE. Tube formation was determined by the length of tube-like structures containing connected cells. LY (20 μM) was used as positive controls for angiogenesis inhibition in this assay. Data are mean ± S.E.M. from three independent experiments. Statistical analysis was performed using one-way ANOVA (*p < 0.05, **p < 0.005, ***p < *0.001*). (**C**) Effect of RCE on cell viability of HUVECs. Viable cells were assayed as described in Materials and Methods. Columns represents mean; bars represents SEM.

Tumor growth and metastasis critically depend on angiogenesis; therefore inhibiting this process would inhibit tumor expansion. We hence used HUVECs plated on Matrigel–coated plates to examine the effect of RCE on the formation of capillary tube *in vitro*. As shown in Fig. 5A,B, treatment of HUVECs with RCE significantly inhibited tube formation when compared with untreated cells. As expected, LY (20 μM), used as positive controls for angiogenesis inhibition in this assay, also inhibited efficiently tube formation (Fig. 5B). RCE-mediated inhibition of tube formation occurred without reduction of cell viability of HUVECs, hence ruling out the possibility that the inhibition of tube formation is a consequence of cell death (Fig. 5C). Thus, our data demonstrates a potent anti-angiogenic activity of *Rhus coriaria*.

**Figure 6:**
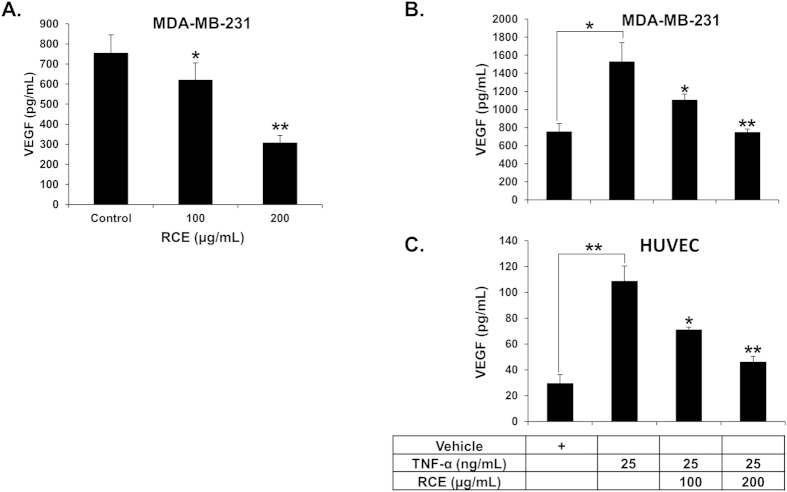
Reduced VEGF secretion in *Rhus coriaria*-treated HUVECs and MDA-MB-231 cells. (**A**) Quantification of basal level of VEGF secretion in conditioned medium from vehicle (0.2% ethanol) or RCE-treated MDA-MB-231 cells. MDA-MB-231 cells were treated with vehicle (ethanol) or the indicated concentrations of RCE for 24 h and then secreted VEGF, in the conditioned medium, was analyzed by ELISA as described in Materials and Methods. (**B**,**C**) Reduction of VEGF secretion in TNF-α induced MDA-MB-231 cells (**B**) and HUVECs (**C**) cultured in presence of vehicle (ethanol) or indicated concentrations of RCE. VEGF secretion was quantified by ELISA as described above. Data represents means ± SEM of three independent experiments. Statistical analysis was performed using one-way ANOVA (*p < 0.05, **p < 0.005, ***p < *0.001*).

VEGF, a pro-angiogenic growth factor, plays an important role in angiogenesis. Therefore, we evaluated the effect of *Rhus coriaria* on the production of VEGF by MDA-MB-231 and HUVECs. Figure 6A shows that treatment with increasing concentrations of RCE markedly reduced the secretion of VEGF by MDA-MB-231 cells. Subsequently, we measured the level of VEGF in MDA-MB-231 cells that were first treated with various concentrations of RCE and then stimulated with TNF-α. We found that the production of VEGF was enhanced in TNF-α-stimulated (1600 pg/mL) compared to vehicle (ethanol)-treated (800 pg/mL) MDA-MB-231 cells (Fig. 6B). However, VEGF production was significantly reduced by RCE in a concentration-dependent manner (Fig. 6B). We have also examined the VEGF production in TNF-α stimulated HUVECs. As expected, HUVEC cultured in the absence of TNF-α produced low level of VEGF (30 pg/mL), while in the presence of TNF-α stimulation, the production level rose to approximately 110 pg/mL (Fig. 6C). Importantly, exposure of HUVECs to RCE also led to a marked concentration-dependent suppression of VEGF production in these cells (Fig. 6C). Altogether, our results suggest that the inhibition of angiogenesis might be a consequence of downregulation of VEGF by RCE.

### *Rhus coriaria* downregulates the expression of TNF-α protein and reduces levels of IL-6, and IL-8 production in breast cancer cells

**Figure 7:**
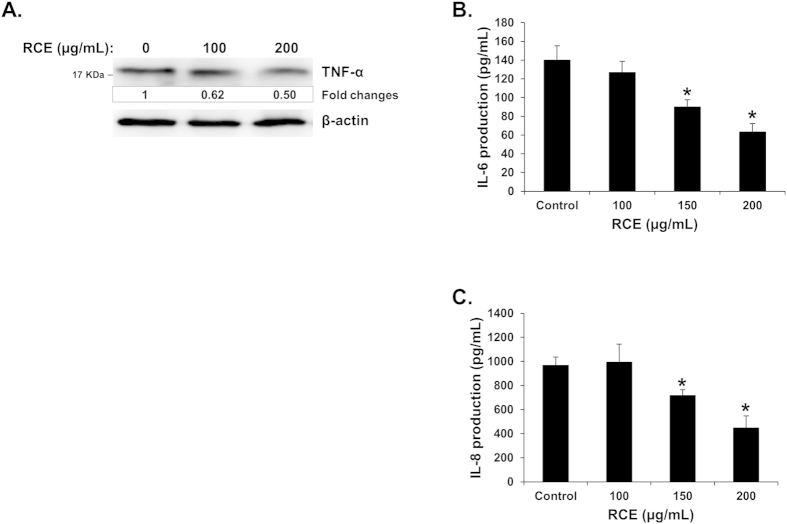
Effects of *Rhus coriaria* on the expression of TNF-α, IL-6 and IL-8 cytokines in MDA-MB-231 cells. (**A**) Western blot quantification of TNF-α protein in RCE-treated MDA-MB-231 cells. MDA-MB-231 cells were treated with vehicle (0.2% ethanol) or indicated concentrations of RCE for 24 h, and then whole-cell extracts were subjected to Western blot analysis for TNF-α and β-actin proteins. Each lane was loaded with 25 μg of whole cell extracts. Loading was normalized to the levels of β-actin. The intensities of the bands were quantified using the ImageJ software (National Institute of Health, USA). (**B**,**C**) Reduction of IL-6 (**B**) and IL-8 (**C**) production in MDA-MB-231 cells. IL-6 and IL-8 production was quantified by ELISA as described in Materials and Methods. Data represents means ± SEM of three independent experiments. Statistical analysis was performed using one-way ANOVA (*p < 0.05, **p < *0.005*).

Several studies reported that the cytokine TNF-α is involved in tumor cell motility and invasion of various cancer including TNBC. Hence, targeting TNF-α should be considered as a therapeutic approach for breast cancer treatment. Because we found that *Rhus coriaria* inhibited cell migration and invasion of MDA-MB-231, we sought to examine the effect of RCE on the expression of TNF-α by measuring the protein level of this cytokine. Figure 7A shows that RCE induced a marked reduction of TNF-α protein in MDA-MB-231 cells.

Next, we have examined the expression level of two other cytokines produced by breast cancers, namely IL-6 and IL-8, which have been shown to increase growth and metastatic potential of breast cancer cells. Interestingly, we found that RCE also significantly reduced the level of both cytokines production in MDA-MB-231 cells in a concentration-dependent manner (Fig. 7B,C).

Hence, our data suggests that downregulation of TNF-α, IL-6 and IL-8 cytokines could account, at least partly, for the inhibition of tumor growth and metastasis mediated by *Rhus coriaria*.

### *Rhus coriaria* attenuates STAT3 activation and inhibits NFκB pathway in MDA-MB-231 breast cancer cells

**Figure 8:**
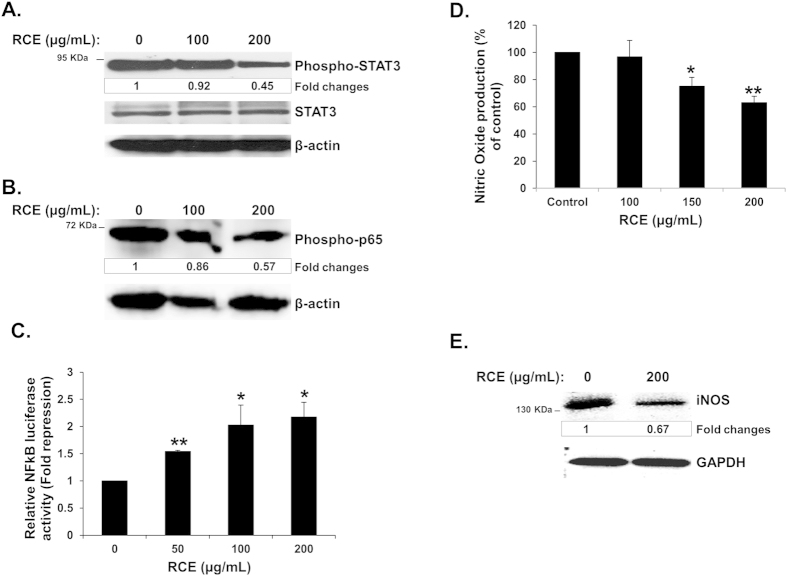
Downregulation of STAT3, NFκB and iNOS and Reduction of Nitric Oxide (NO) production in *Rhus coriaria*-treated MDA-MB-231 cells. (**A**) Concentration-dependent decrease of phospho-STAT3 in RCE-treated MDA-MB-231 cells. MDA-MB-231 cells were treated with vehicle (0.2% ethanol) or indicated concentrations of RCE for 24 h, then whole-cell extracts were subjected to Western blot analysis for the phosphorylated and non-phosphorylated form of STAT3 and for β-actin (loading control). (**B**,**C**) Inhibition of the NFκB signaling pathway by RCE. (**B**) Western blot analysis showing a concentration-dependent decrease of phospho-p65 (NFκB) in MDA-MB-231 cells in response to RCE treatment. (**C**) Inhibition, by RCE, of NFκB transcriptional activity in MDA-MB-231 cells. MDA-MB-231 cells were transfected with the pGL4.32[luc2P/NFκB-RE/Hygro] expression plasmid and luciferase activity was measured 18 h post-transfection as described in Materials and Methods. Columns represents mean; bars represents SEM of three independent experiments. Statistical analysis was performed using one-way ANOVA (*p < 0.05, **p < 0.005, ***p < 0.001). (**D**–**E**) Reduction of Nitric Oxide (NO) production and downregulation of iNOS in RCE-treated MDA-MB-231 cells. (**D**) Quantification of NO levels in RCE-treated MDA-MB-231 cells. Cells were treated with vehicle (0.2% ethanol) or indicated concentrations of RCE for 24 h and Nitrate/Nitrite production was quantified as described in Materials and Methods. Statistical analysis was performed using one-way ANOVA (*p < 0.05, **p < 0.005, ***p < 0.001). Data represents means ± SEM of three independent experiments carried out in triplicate. (**E**) Western blot quantification of iNOS in cells treated without or with the indicated concentration of RCE. For western blot, each lane was loaded with 25 μg of whole cell extracts. Loading was normalized to the levels of β-actin.The intensities of the bands were quantified using the ImageJ software (National Institute of Health, USA).

STAT3, activated by phosphorylation, is considered as mediator of tumorigenesis because of its role in promoting cellular proliferation, resistance to apoptosis, tumor angiogenesis, invasion, and migration of cancer cells^25^. Hence, STAT3 is widely recognized as a potential drug target for cancer therapy. To investigate whether *Rhus coriaria* affects the activation of STAT3 signaling in breast cancer cells, we analyzed the level of pSTAT3 in RCE-treated MDA-MB-231 cells. We found that RCE markedly reduced STAT3 phosphorylation, in concentration dependent-manner in MDA-MB-231 cells (Fig. 8A). This result suggests that the RCE-mediated effect on cell proliferation, migration and invasion in breast cancer involves the inhibition of STAT3 signaling in breast cancer cells.

The NFκB signaling pathway is also known to regulate the expression of various genes involved in tumor cells invasion. To investigate whether RCE could affect this pathway, we first examined, by Western blotting, the phosphorylation status of phospho-p65 in RCE-treated MDA-MB-231 cells. We found that RCE drastically inhibited the phosphorylation of p65 (Fig. 8B). Next, we decided to measure the ability of RCE to inhibit NFκB transcriptional activity. Toward this, MDA-MB-231 cells were transiently transfected with an NFκB reporter expression vector as described in materials and methods. As it is shown in Fig. 8C, RCE repressed NFκB-dependent transcription of the luciferase reporter in a concentration-dependent manner.

Taken together, our data clearly indicate that RCE exerts its effects on breast cancer cell proliferation, migration and invasion at least partly through an inhibition of two key signaling pathways, namely STAT3 and NFκB, known to regulate several processes such as tumor growth and metastasis in breast cancer.

### Reduced Nitric Oxide production in *Rhus coriaria*-treated MDA-MB-231 cells

Because nitric oxide (NO) signaling has also been shown to promote breast tumor growth and metastasis by altering the expression of genes implicated in cellular migration, invasion and angiogenesis^26^,^27^,^28^, we examined the effect of RCE on the NO production in MDA-MB-231 cells. Results in Fig. 8D clearly show that RCE decreased NO production in a concentration-dependent manner in MDA-MB-231 cells.

Inducible nitric oxide synthase (iNOS) is one of the three enzymes responsible for the NO synthesis and its overexpression is documented in breast cancer^29^,^30^,^31^,^32^. To further understand the mechanism of RCE inhibition of NO production, we examined the expression level of iNOS in RCE-treated MDA-MB-231 cells. Indeed, we found that treatment of these cells with RCE led to a decrease in the protein level of iNOS compared to untreated cells (Fig. 8E). Taken together, our results suggest that RCE could also exert its anti-metastatic effect by modulating the level of iNOS and consequently of NO production in breast cancer cells.

### *Rhus coriaria* inhibits tumor growth and metastasis in chick embryo tumor growth and metastasis assay

**Figure 9:**
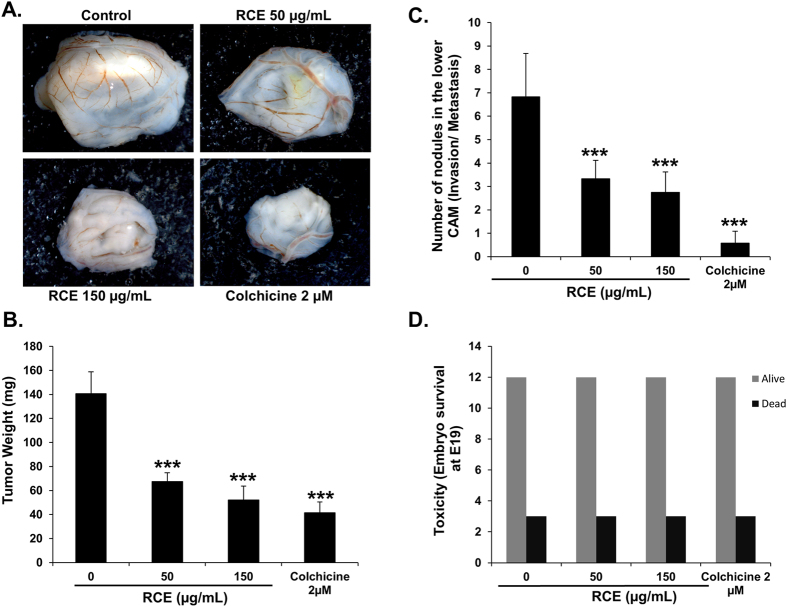
Anti-tumor growth and anti-metastatic activity of *Rhus coriaria* on breast tumor in chick embryo chorioallantoic membrane model system. (**A**) MDA-MB-231 (1 × 10^6^) cells were grafted on the CAM of 10 day (E10) chick embryo. Tumors were treated every 48 h with RCE as described in Materials and Methods. At E19, tumors were collected and weighted. (**B**) Quantification of tumor weight in vehicle (ethanol), colchicine and indicated concentrations of RCE-treated chick embryo. (**C**) Anti-metastatic effect of *Rhus coriaria*. Quantification of nodules observed in the lower CAM of chick embryo treated with vehicle (ethanol), colchicine or indicated concentrations of RCE. Columns represents mean; bars represents SEM. ***significantly different at *p* < 0.0005. (**D**) Toxicity screening of RCE on chick embryos. Toxicity was evaluated by comparing the number of dead embryos in control, colchicine- and RCE-treated embryos.

Finally, we tested the effect of *Rhus coriaria* on tumor growth *in vivo* by using the chick embryo model. MDA-MB-231 cells were grafted on the chorioallantoic membrane (CAM) and formed tumors were treated every 48 h with vehicle (ethanol), colchicine (2 μM) or increased concentrations of RCE (50 and 150 μg/mL). At E 19, tumors were recovered from the upper CAM and weighted. We found that RCE significantly inhibited tumor growth compared with the vehicle (ethanol) treatment even at low concentration. In fact, concentrations of only 50 μg/mL RCE led to reduced tumor growth by 52% and a concentration of 150 μg/mL led to a further reduction (63%) (Fig. 9A,B). We have also assessed for the ability of RCE to inhibit metastasis by counting the number of nodules in the lower CAM in vehicle (ethanol), colchicine- and RCE-treated tumors. An average of 6.83 ± 1.85 nodules were counted in the lower CAM of vehicle (ethanol)-treated chick embryo, while an average of 3.33 ± 0.78 and 2.75 ± 0.87 nodules only were counted in 50 and 150 μg/mL RCE-treated embryo (Fig. 9C), respectively. Altogether, our data clearly demonstrates that RCE could efficiently inhibit breast tumor growth and metastasis *in vivo*. Toxicity was also evaluated by comparing the number of dead embryos in control, colchicine- and RCE-treated embryos. At the end of the experiment (E19), RCE showed no cytotoxicity as there was no difference in the number of surviving embryo in control and *Rhus coriaria* treatment (Fig. 9D).

## Discussion

Cancer metastasis requires several crucial interrelated events such as cancer cell adhesion, proteolytic degradation of and migration through the extra cellular matrix as well as angiogenesis. Current cancer treatment drugs aim at blocking cell cycle progression, inducing cell death and/or inhibiting tumor invasion and angiogenesis. Interestingly, cancer chemoprevention targeting these events has been reported for several natural compounds. Still, there is a growing interest in combination therapy using multiple anticancer drugs targeting several targets/pathways. Our findings demonstrate for the first time that *Rhus coriaria*, at non-cytotoxic concentrations, exerts a potent anti-metastatic and anti-tumor growth activities against TNBC. Indeed, we report that *Rhus coriari*a inhibited the migration and invasion of MDA-MB-231 cells, blocked their adhesion to fibronectin, to HUVECs and to lung microvascular endothelial (HMVEC-L) and inhibited their transendothelial migration through TNF-α-activated HUVECs. Furthermore, we found that *Rhus coriaria* inhibited angiogenesis, reduced VEGF production in both MDA-MB-231 and HUVECs and downregulated MMP-9, PgE2, TNF-α, IL-6 and IL-8. Interestingly, our investigation revealed that NFκB, STAT3 and Nitric Oxide (NO) pathways were inhibited in MDA-MB-231 in response to *Rhus coriaria.* Moreover, we demonstrated that *Rhus coriaria* significantly inhibited tumor growth and metastasis *in vivo*.

The ability of cancer cells to adhere to components of the extracellular matrix (ECM) is a pre-requisite process for cancer cell migration and thus represents one of the key steps in cancer metastasis. Indeed, it has been shown that the interaction of fibronectin, a component of the ECM, with specific cell surface receptors belonging to the integrin family, enhances the metastatic potential of breast cancer cells^33^. Hence, blocking the fibronectin-integrin would significantly diminish cell adhesion to ECM and consequently would block cell migration. Our data demonstrated that non-cytotoxic concentrations of *Rhus coriaria* markedly inhibited the adhesion of MDA-MB-231 to fibronectin as well as their migration. This inhibition may account, at least partly, for the anti-metastatic potential of *Rhus coriaria* on breast cancer cells. One may then hypothesize that *Rhus coriaria* might be a valuable source of potentially new compound(s) that can interfere with the adhesion of cancer cells to the ECM.

Dissemination of cancer cells from the primary tumor is another crucial event in the process of cancer invasion and metastasis. This process involves degradation of the ECM through many proteases, of which MMP-9 appears to play a key role^34^,^35^. Indeed, increased expression of MMPs correlates with increased growth, aggressiveness and metastatic potential of breast cancer cells^36^. Therefore, inhibiting these proteases is an important approach in the fight against breast cancer. Here, we clearly demonstrate that *Rhus coriaria* exerts its anti-invasive effects by decreasing MMP-9 levels and consequently reducing ECM degradation.

COX-2-derived prostaglandins are well known to promote proliferation, migration, invasion, and metastasis of cancer cells. One of the prostanoids that plays a vital role in malignancy is prostaglandin E_2_ (PgE2). A study by Singh and collaborators showed that COX-2 expression and the production of PgE2 in breast tumors stimulate bone metastasis in both mouse models and patients with breast cancer^37^. Downregulation of PgE2 production through inhibition of COX-2 in malignant breast cancer cells resulted in a significant attenuation of migration and invasion in MDA-MB-231 cells^38^. The development of agents that lower cellular levels of PgE2 might be very useful for treatment and prevention of breast cancer. Interestingly, we found that *Rhus coriaria* significantly reduced PgE2 production in MDA-MB-231 cells. Thus, our findings suggest that downregulation of PgE2 production could be one of the mechanisms through which *Rhus coriaria* inhibits breast cancer tumorigenesis. As such, *Rhus coriaria* may turn out to be an important source for new inhibitors of COX-2/PgE2.

Angiogenesis, a process by which new blood vessels form, is crucial for tumor growth and metastasis. Preventing this process would ultimately inhibit both tumor growth and metastasis^39^. Here, we showed that *Rhus coriaria* markedly blocked angiogenesis by inhibiting tube formation by HUVECs. One possible way to block angiogenesis is to target pro-angiogenic factors secreted by tumor cells such as VEGF. As matter of fact, VEGF is considered as a major pro-angiogenic protein expressed in 60% of breast cancer patients at the time of first diagnosis^40^. Importantly, we found that RCE significantly reduced the production of VEGF in both HUVECs and MDA-MB-231 cells and thus suggesting that one possible mechanism through which *Rhus coriaria* inhibits TNBC tumor growth is by blocking the process of angiogenesis.

Accumulating evidence suggests a strong association between inflammation and cancer progression^41^. Increased levels of inflammatory cytokines, such as IL-6, IL-8, and TNF-α, are known to promote migration, invasion and metastasis of various types of cancer including breast cancer^42^. Several studies identified IL-6, IL-8 and TNF-α as important factors of poor prognosis in TNBC, given their role in promoting invasion and metastasis^43^,^44^,^45^. Hence, inhibition of these signaling pathways offers a promising strategy in targeting TNBC. Recently, Hartman and collaborators reported that growth of TNBC relies upon coordinate autocrine expression of IL-6 and IL-8. Moreover, concurrent inhibition of these two cytokines inhibited colony formation *in vitro* and tumor growth *in vivo* of TNBC cells^46^. Similarly, inhibition of MDA-MB-231 tumor xenografts by ulinastatin and docetaxel was associated with downregulation of the expression of IL-6, IL-8 and TNF- α^47^. It is also known that TNF-α increasesIL-6 production^48^. Interestingly, here we showed that RCE inhibited both TNF-α and IL-6 production in MDA-MB-231 cells. It is then possible to postulate that RCE inhibition of IL-6 occurs by virtue of its ability to suppress the TNF signaling route. Moreover, we also showed that RCE could efficiently inhibit IL-8 production. Taken together, our data strongly suggests that one possible mechanism through which RCE inhibits invasion and tumor growth involves inhibition of IL-6, IL-8 and TNF-α pathways.

Transcription factors including NFκB and STAT3 play crucial roles in transmitting signals of inflammatory cytokines to the nucleus^49^. The NFκB signaling pathway, which can be activated by TNF-α, is known to activate the expression of various genes involved in the process of tumor metastasis and growth and its inactivation suppress metastasis of breast cancer cells. Moreover, inactivation of NFκB in breast cancer cells inhibits the expression of many target genes involved in tumor metastasis and tumor growth such as MMP-9^50^, VEGF^50^, IL-6^46^ and IL-8^46^.

In the present work, we demonstrated that *Rhus coriaria* inhibited the NFκB signaling through downregulation of phospho-p65 as well as its downstream targets (MMP-9, VEGF, IL-6 and IL-8), all of which are involved in tumor metastasis. It is noteworthy to mention that *Rhus coriaria* also efficiently inhibited TNF-α in MDA-MB-231 cells. It appears then that inhibition of NFκB could account, although not solely, for the anti-metastatic effects of *Rhus coriaria.* We can hypothesize that one possible mechanism by which *Rhus coriaria* exerts its anti-metastatic and anti-tumor growth of TNBC involves the downregulation of TNF-α.

Constitutive activation of STAT3 was described in various types of cancers including breast cancer. The critical role of STAT3 in tumor cell survival, proliferation, tumor cell invasion, metastasis and angiogenesis is well-established and thus targeting STAT3 for therapy assaults cancer on multiple fronts^51^,^52^. A possible mechanism through which STAT3 is hyperactivated in tumors involves the autocrine production of IL-6 from tumor cells^49^. Indeed, in cancer inflammatory environment, IL-6 induces dimerization of IL-6 receptors, leading to activation of STAT3^53^. This increased activation can then promote cancer progression and metastasis. Interestingly, in MDA-MB-231, STAT3 can itself increase the expression of IL-6 for further activation of STAT3^54^. Interestingly, we found that *Rhus coriaria* inhibited STAT3 phosphorylation in a concentration-dependent manner in MDA-MB-231 cells, thus suggesting that STAT3 inactivation might contribute to the anti-cancer effect of *Rhus coria*ria. Most importantly, IL-6 production was also reduced in response to *Rhus coriaria*. Thus, our data suggest that reduction of IL-6 production might contribute, at least partly, to the inhibition of STAT3 in TNBC.

Nitric Oxide (NO) is a signaling molecule that regulates several physiological processes such as vasodilatation, cell migration, immune reactions and apoptosis^28^. Interestingly, NO can also promote or inhibit tumor progression and metastasis. The pro- or anti-tumorigenic activities of NO have been related to the p53 status^55^,^56^. For example, NO-mediated apoptosis in leukemia cells requires wild-type p53^57^. On the other hand, carcinoma cells with mutant p53 and expressing iNOS/NOS2 have accelerated tumor growth and increased VEGF production^30^. Recent studies showed that increased iNOS/NOS2 and consequently NO production, predicted poor survival in patients with TNBC^30^. Moreover, exposure to NO enhanced cell motility and invasion of Estrogen Receptor negative cells^30^. Selective iNOS inhibitor (1400W) diminished cell proliferation and migration and inhibited tumor growth of TNBC cells^58^. It appears then that discovery of new inhibitors targeting NO production may be particularly efficacious against TNBC patients. Here, we showed that *Rhus coriaria* significantly downregulated iNOS and consequently decreased NO production in TNBC. Taken together, these data indicate that *Rhus coriaria* might also exert its anti-metastatic effect by targeting the NO signaling pathway.

In conclusion, our data clearly demonstrate that *Rhus coriaria* exerts a potent anti-angiogenic, anti-metastatic and anti-tumor growth effects on TNBC by targeting multiple key pathways employed by TNBC to acquire a rather aggressive phenotype. Our findings provide the first instance of a potential role for *Rhus coriaria* as an anti-breast cancer agent and certainly deserves more attention for further explorations to identify novel effective therapeutic compound(s) against TNBC.

## Additional Information

**How to cite this article**: El Hasasna, H. *et al. Rhus coriaria* suppresses angiogenesis, metastasis and tumor growth of breast cancer through inhibition of STAT3, NF_κ_B and nitric oxide pathways. *Sci. Rep.* **6**, 21144; doi: 10.1038/srep21144 (2016).

## Supplementary Material

Supplementary Information

## References

[b1] WHO | Cancer. *WHO (World Health Organization)* (2014). at http://www.who.int/mediacentre/factsheets/fs297/en/.

[b2] Mudvari P., Ohshiro K., Nair V., Horvath A. & Kumar R. Genomic insights into triple-negative and HER2-positive breast cancers using isogenic model systems. PloS One 8, e74993 (2013). 24086418 10.1371/journal.pone.0074993 PMC3781103

[b3] Kashiwagi S. . Significance of E-cadherin expression in triple-negative breast cancer. Br. J. Cancer 103, 249–255 (2010). 20551954 10.1038/sj.bjc.6605735 PMC2906732

[b4] Libério M. S., Joanitti G. A., Fontes W. & Castro M. S. Anticancer peptides and proteins: a panoramic view. Protein Pept. Lett. 20, 380–391 (2013). 23016586 10.2174/092986613805290435

[b5] Dent R. . Triple-negative breast cancer: clinical features and patterns of recurrence. Clin. Cancer Res. Off. J. Am. Assoc. Cancer Res. 13, 4429–4434 (2007). 10.1158/1078-0432.CCR-06-3045 17671126

[b6] Irvin W. J. & Carey L. A. What is triple-negative breast cancer? Eur. J. Cancer Oxf. Engl. 1990 44, 2799–2805 (2008). 10.1016/j.ejca.2008.09.034 19008097

[b7] Cassidy A. Are herbal remedies and dietary supplements safe and effective for breast cancer patients? Breast Cancer Res. BCR 5, 300–302 (2003). 14580245 10.1186/bcr724 PMC314418

[b8] Singh A., Saharan V. A. & Bhandari A. Pharmacognostic standardization with various plant parts of Desmostachya bipinnata. Pharm. Biol. 52, 298–307 (2014). 24107271 10.3109/13880209.2013.834367

[b9] Newman D. J. & Cragg G. M. Natural products as sources of new drugs over the 30 years from 1981 to 2010. J. Nat. Prod. 75, 311–335 (2012). 22316239 10.1021/np200906s PMC3721181

[b10] Rayne S. & Mazza G. Biological activities of extracts from sumac (Rhus spp.): a review. Plant Foods Hum. Nutr. Dordr. Neth. 62, 165–175 (2007). 10.1007/s11130-007-0058-4 17909971

[b11] Zargham H. & Zargham R. Tannin extracted from Sumac inhibits vascular smooth muscle cell migration. McGill J. Med. MJM Int. Forum Adv. Med. Sci. Stud. 11, 119–123 (2008). PMC2582678 19148309

[b12] Abu-Reidah I. M., Ali-Shtayeh M. S., Jamous R. M., Arráez-Román D. & Segura-Carretero A. HPLC-DAD-ESI-MS/MS screening of bioactive components from *Rhus coriaria* L. (Sumac) fruits. Food Chem. 166, 179–191 (2015). 25053044 10.1016/j.foodchem.2014.06.011

[b13] Kizil S. & Turk M. Microelement contents and fatty acid compositions of *Rhus coriaria* L. and Pistacia terebinthus L. fruits spread commonly in the south eastern Anatolia region of Turkey. Nat. Prod. Res. 24, 92–98 (2010). 20013479 10.1080/14786410903132555

[b14] Bursal E. & Köksal E. Evaluation of reducing power and radical scavenging activities of water and ethanol extracts from sumac (*Rhus coriaria* L.). Food Res. Int. 44, 2217–2221 (2011).

[b15] Pourahmad J., Eskandari M. R., Shakibaei R. & Kamalinejad M. A search for hepatoprotective activity of aqueous extract of *Rhus coriaria* L. against oxidative stress cytotoxicity. Food Chem. Toxicol. Int. J. Publ. Br. Ind. Biol. Res. Assoc. 48, 854–858 (2010). 10.1016/j.fct.2009.12.021 20036300

[b16] Shabana M. M., El Sayed A. M., Yousif M. F., El Sayed A. M. & Sleem A. A. Bioactive constituents from Harpephyllum caffrum Bernh. and *Rhus coriaria* L. Pharmacogn. Mag. 7, 298–306 (2011). 22262932 10.4103/0973-1296.90410 PMC3261063

[b17] Capcarova M. . Effects of dietary inclusion of *Rhus coriaria* on internal milieu of rabbits. J. Anim. Physiol. Anim. Nutr. 96, 459–465 (2012). 10.1111/j.1439-0396.2011.01164.x 21585564

[b18] El Hasasna H. . *Rhus coriaria* induces senescence and autophagic cell death in breast cancer cells through a mechanism involving p38 and ERK1/2 activation. Sci. Rep. 5, 13013 (2015). 26263881 10.1038/srep13013 PMC4532997

[b19] Prudent R. . Azaindole derivatives are inhibitors of microtubule dynamics, with anti-cancer and anti-angiogenic activities. Br. J. Pharmacol. 168, 673–685 (2013). 23004938 10.1111/j.1476-5381.2012.02230.x PMC3579287

[b20] Al Dhaheri Y. . Anti-metastatic and anti-tumor growth effects of Origanum majorana on highly metastatic human breast cancer cells: inhibition of NFκB signaling and reduction of nitric oxide production. PloS One 8, e68808 (2013). 23874773 10.1371/journal.pone.0068808 PMC3707896

[b21] Zen K. . CD44v4 is a major E-selectin ligand that mediates breast cancer cell transendothelial migration. PloS One 3, e1826 (2008). 18350162 10.1371/journal.pone.0001826 PMC2265551

[b22] Green C. E. . Chemoattractant Signaling between Tumor Cells and Macrophages Regulates Cancer Cell Migration, Metastasis and Neovascularization. PLoS ONE 4, e6713 (2009). 19696929 10.1371/journal.pone.0006713 PMC2725301

[b23] Timoshenko A. V., Xu G., Chakrabarti S., Lala P. K. & Chakraborty C. Role of prostaglandin E2 receptors in migration of murine and human breast cancer cells. Exp. Cell Res. 289, 265–274 (2003). 14499627 10.1016/s0014-4827(03)00269-6

[b24] Zheng H. . Downregulation of COX-2 and CYP 4A signaling by isoliquiritigenin inhibits human breast cancer metastasis through preventing anoikis resistance, migration and invasion. Toxicol. Appl. Pharmacol. 280, 10–20 (2014). 25094029 10.1016/j.taap.2014.07.018

[b25] Kamran M. Z., Patil P. & Gude R. P. Role of STAT3 in cancer metastasis and translational advances. BioMed Res. Int. 2013, 421821 (2013). 24199193 10.1155/2013/421821 PMC3807846

[b26] Jadeski L. C., Hum K. O., Chakraborty C. & Lala P. K. Nitric oxide promotes murine mammary tumour growth and metastasis by stimulating tumour cell migration, invasiveness and angiogenesis. Int. J. Cancer J. Int. Cancer 86, 30–39 (2000). 10.1002/(sici)1097-0215(20000401)86:1<30::aid-ijc5>3.0.co;2-i 10728591

[b27] Vakkala M. . Inducible nitric oxide synthase expression, apoptosis, and angiogenesis in *in situ* and invasive breast carcinomas. Clin. Cancer Res. Off. J. Am. Assoc. Cancer Res. 6, 2408–2416 (2000). 10873093

[b28] Switzer C. H. . Nitric oxide and protein phosphatase 2A provide novel therapeutic opportunities in ER-negative breast cancer. Trends Pharmacol. Sci. 32, 644–651 (2011). 21893353 10.1016/j.tips.2011.07.001 PMC3380363

[b29] Bulut A. S. . Significance of inducible nitric oxide synthase expression in benign and malignant breast epithelium: an immunohistochemical study of 151 cases. Virchows Arch. Int. J. Pathol. 447, 24–30 (2005). 10.1007/s00428-005-1250-2 15947943

[b30] Glynn S. A. . Increased NOS2 predicts poor survival in estrogen receptor-negative breast cancer patients. J. Clin. Invest. 120, 3843–3854 (2010). 20978357 10.1172/JCI42059 PMC2964971

[b31] Loibl S. . The role of early expression of inducible nitric oxide synthase in human breast cancer. Eur. J. Cancer Oxf. Engl. 1990 41, 265–271 (2005). 10.1016/j.ejca.2004.07.010 15661552

[b32] Thomsen L. L. . Nitric oxide synthase activity in human breast cancer. Br. J. Cancer 72, 41–44 (1995). 7541238 10.1038/bjc.1995.274 PMC2034139

[b33] Bartsch J. E., Staren E. D. & Appert H. E. Adhesion and migration of extracellular matrix-stimulated breast cancer. J. Surg. Res. 110, 287–294 (2003). 12697413 10.1016/s0022-4804(03)00004-0

[b34] Liotta L. A. . Metastatic potential correlates with enzymatic degradation of basement membrane collagen. Nature 284, 67–68 (1980). 6243750 10.1038/284067a0

[b35] Gialeli C., Theocharis A. D. & Karamanos N. K. Roles of matrix metalloproteinases in cancer progression and their pharmacological targeting. FEBS J. 278, 16–27 (2011). 21087457 10.1111/j.1742-4658.2010.07919.x

[b36] Bachmeier B. E., Nerlich A. G., Lichtinghagen R. & Sommerhoff C. P. Matrix metalloproteinases (MMPs) in breast cancer cell lines of different tumorigenicity. Anticancer Res. 21, 3821–3828 (2001). 11911253

[b37] Singh B. . COX-2 involvement in breast cancer metastasis to bone. Oncogene 26, 3789–3796 (2007). 17213821 10.1038/sj.onc.1210154

[b38] Kim M.-J. . NDRG2 controls COX-2/PGE_2_-mediated breast cancer cell migration and invasion. Mol. Cells 37, 759–765 (2014). 25256221 10.14348/molcells.2014.0232 PMC4213768

[b39] Kerbel R. & Folkman J. Clinical translation of angiogenesis inhibitors. Nat. Rev. Cancer 2, 727–739 (2002). 12360276 10.1038/nrc905

[b40] Relf M. . Expression of the angiogenic factors vascular endothelial cell growth factor, acidic and basic fibroblast growth factor, tumor growth factor beta-1, platelet-derived endothelial cell growth factor, placenta growth factor, and pleiotrophin in human primary breast cancer and its relation to angiogenesis. Cancer Res. 57, 963–969 (1997). 9041202

[b41] Mantovani A. Cancer: inflammation by remote control. Nature 435, 752–753 (2005). 15944689 10.1038/435752a

[b42] Aggarwal B. B., Shishodia S., Sandur S. K., Pandey M. K. & Sethi G. Inflammation and cancer: how hot is the link? Biochem. Pharmacol. 72, 1605–1621 (2006). 16889756 10.1016/j.bcp.2006.06.029

[b43] Salgado R. . Circulating interleukin-6 predicts survival in patients with metastatic breast cancer. Int. J. Cancer J. Int. Cancer 103, 642–646 (2003). 10.1002/ijc.10833 12494472

[b44] Rody A. . A clinically relevant gene signature in triple negative and basal-like breast cancer. Breast Cancer Res. BCR 13, R97 (2011). 21978456 10.1186/bcr3035 PMC3262210

[b45] Balkwill F. TNF-alpha in promotion and progression of cancer. Cancer Metastasis Rev. 25, 409–416 (2006). 16951987 10.1007/s10555-006-9005-3

[b46] Hartman Z. C. . Growth of triple-negative breast cancer cells relies upon coordinate autocrine expression of the proinflammatory cytokines IL-6 and IL-8. Cancer Res. 73, 3470–3480 (2013). 23633491 10.1158/0008-5472.CAN-12-4524-T PMC3853111

[b47] Zhao X., Sun X., Gao F., Luo J. & Sun Z. Effects of ulinastatin and docataxel on breast tumor growth and expression of IL-6, IL-8, and TNF-α. J. Exp. Clin. Cancer Res. CR 30, 22 (2011). 21345194 10.1186/1756-9966-30-22 PMC3050767

[b48] Suarez-Cuervo C., Harris K. W., Kallman L., Väänänen H. K. & Selander K. S. Tumor necrosis factor-alpha induces interleukin-6 production via extracellular-regulated kinase 1 activation in breast cancer cells. Breast Cancer Res. Treat. 80, 71–78 (2003). 12889600 10.1023/a:1024443303436

[b49] Yoshimura A. Signal transduction of inflammatory cytokines and tumor development. Cancer Sci. 97, 439–447 (2006). 16734720 10.1111/j.1349-7006.2006.00197.x PMC11159428

[b50] Kortylewski M., Jove R. & Yu H. Targeting STAT3 affects melanoma on multiple fronts. Cancer Metastasis Rev. 24, 315–327 (2005). 15986140 10.1007/s10555-005-1580-1

[b51] Rivat C. . Implication of STAT3 signaling in human colonic cancer cells during intestinal trefoil factor 3 (TFF3)–and vascular endothelial growth factor-mediated cellular invasion and tumor growth. Cancer Res. 65, 195–202 (2005). 15665295

[b52] Huang C. . Effects of IL-6 and AG490 on regulation of Stat3 signaling pathway and invasion of human pancreatic cancer cells *in vitro*. J. Exp. Clin. Cancer Res. CR 29, 51 (2010). 20482858 10.1186/1756-9966-29-51 PMC2883975

[b53] Grivennikov S. I. & Karin M. Dangerous liaisons: STAT3 and NF-kappaB collaboration and crosstalk in cancer. Cytokine Growth Factor Rev. 21, 11–19 (2010). 20018552 10.1016/j.cytogfr.2009.11.005 PMC2834864

[b54] Nizamutdinova I. T. . Tanshinone I suppresses growth and invasion of human breast cancer cells, MDA-MB-231, through regulation of adhesion molecules. Carcinogenesis 29, 1885–1892 (2008). 18586687 10.1093/carcin/bgn151

[b55] Muntané J. & la Mata M. D. Nitric oxide and cancer. World J. Hepatol. 2, 337–344 (2010). 21161018 10.4254/wjh.v2.i9.337 PMC2999298

[b56] Wang C., Trudel L. J., Wogan G. N. & Deen W. M. Thresholds of nitric oxide-mediated toxicity in human lymphoblastoid cells. Chem. Res. Toxicol. 16, 1004–1013 (2003). 12924928 10.1021/tx0340448

[b57] Ambs S. . p53 and vascular endothelial growth factor regulate tumor growth of NOS2-expressing human carcinoma cells. Nat. Med. 4, 1371–1376 (1998). 9846573 10.1038/3957

[b58] Granados-Principal S. . Inhibition of iNOS as a novel effective targeted therapy against triple-negative breast cancer. Breast Cancer Res. BCR 17, 25 (2015). 25849745 10.1186/s13058-015-0527-x PMC4384389

