# Supplementary figures and images for: Rhus coriaria suppresses angiogenesis, metastasis and tumor growth of breast cancer through inhibition of STAT3, NFκB and nitric oxide pathways
Source: Sci Rep. 2016 Feb 18;6:21144. doi: 10.1038/srep21144 (PMC4758048; doi:10.1038/srep21144)

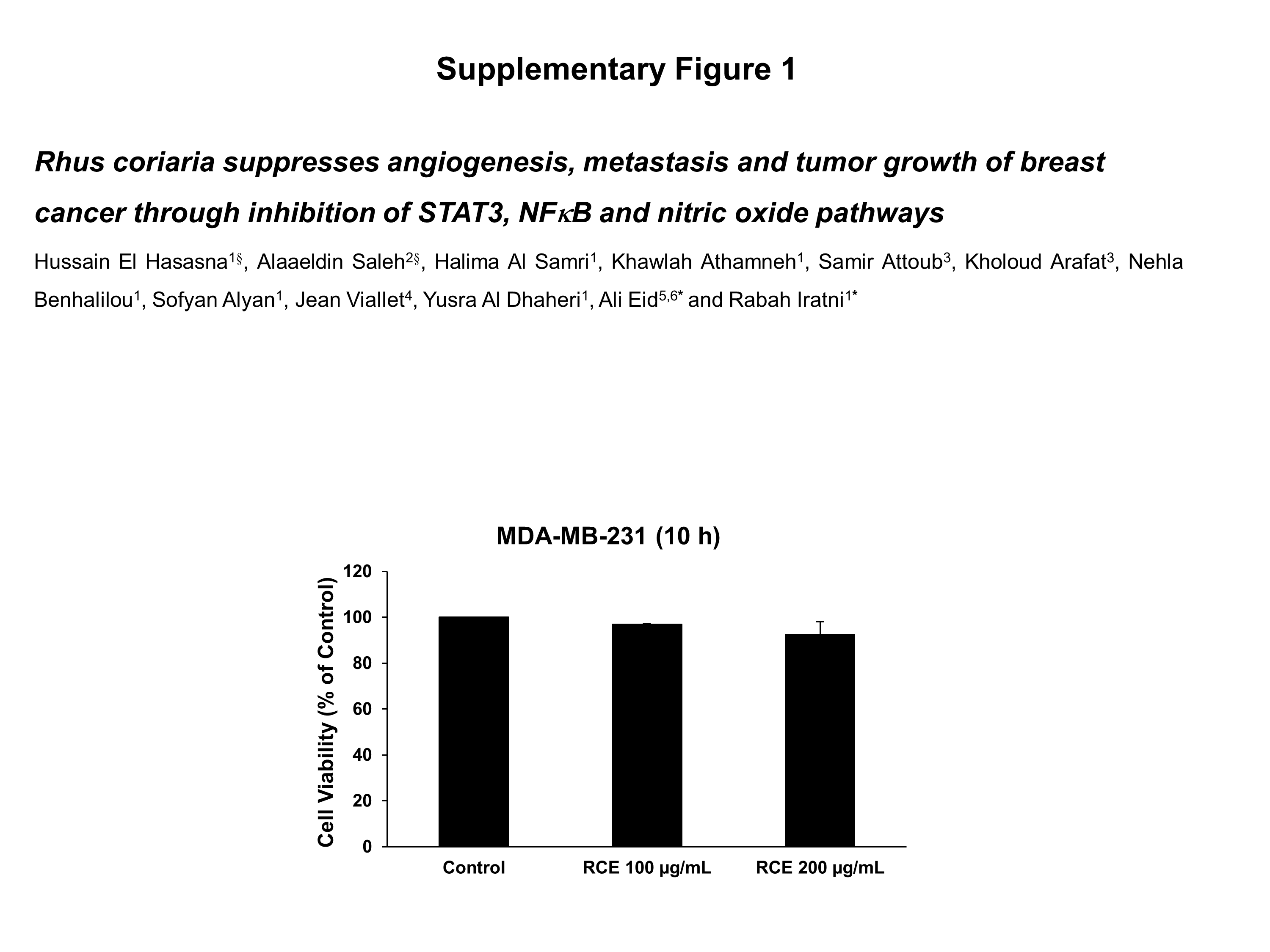

Supplement: Supplementary Information [file srep21144-s1.tiff]
